# Extent and ecological consequences of hunting in Central African rainforests in the twenty-first century

**DOI:** 10.1098/rstb.2012.0303

**Published:** 2013-09-05

**Authors:** K. A. Abernethy, L. Coad, G. Taylor, M. E. Lee, F. Maisels

**Affiliations:** 1 African Forest Ecology Group, School of Natural Sciences, University of Stirling, Stirling, UK; 2 School of Geography and Environmental Planning, University of Queensland, St Lucia Campus, Brisbane, Australia; 3 Environmental Change Institute, School of Geography, University of Oxford, Oxford, UK; 4 Wildlife Conservation Research Unit (WildCRU), University of Oxford, Oxford, UK; 5 Wildlife Conservation Society, Bronx, NY, USA

**Keywords:** Central Africa, hunting, future, wildlife, land-use change, ecological function

## Abstract

Humans have hunted wildlife in Central Africa for millennia. Today, however, many species are being rapidly extirpated and sanctuaries for wildlife are dwindling. Almost all Central Africa's forests are now accessible to hunters. Drastic declines of large mammals have been caused in the past 20 years by the commercial trade for meat or ivory. We review a growing body of empirical data which shows that trophic webs are significantly disrupted in the region, with knock-on effects for other ecological functions, including seed dispersal and forest regeneration. Plausible scenarios for land-use change indicate that increasing extraction pressure on Central African forests is likely to usher in new worker populations and to intensify the hunting impacts and trophic cascade disruption already in progress, unless serious efforts are made for hunting regulation. The profound ecological changes initiated by hunting will not mitigate and may even exacerbate the predicted effects of climate change for the region. We hypothesize that, in the near future, the trophic changes brought about by hunting will have a larger and more rapid impact on Central African rainforest structure and function than the direct impacts of climate change on the vegetation. Immediate hunting regulation is vital for the survival of the Central African rainforest ecosystem.

## 1. Introduction

Hunting is a ubiquitous part of daily life in rural Central Africa. Wild meat is part of the village subsistence economy, and commercial wildlife hunting—practised in Central Africa for at least two millennia—continues today [1,2]. Contemporary illegal wildlife trade, now one of the three most important types of crime on the planet [3], uses village hunters to secure tusks, meat and skins, but an increasing number of commercial hunters, using heavier-calibre weapons than those available to villagers, and particularly targeting ivory-bearing elephants, are also hunting in the region; meat and ivory then pass via highly organized trade chains to their destinations in the cities of the region and overseas [4–7]. The ecological consequences of wildlife hunting in the tropics are far-reaching, with knock-on effects disrupting ecological function over large areas and in key ecosystems [8–10].

Human shaping of the rainforest environment is a significant evolutionary force for the biome [1,11], and African rainforest species, unlike those endemic to other continents, have evolved in the constant presence of hominid hunters and their ancestors [12]. However, modern human hunting pressure, driven largely by increasing commercial trade, has resulted in the local extirpation of many larger African rainforest mammals [13–17] and is changing wildlife assemblages and species interactions [18,19]. Overhunting of wildlife is highly associated with loss of ecological integrity in tropical forests [20] and significant, deleterious trophic cascades in other ecosystems [9,10], and similar changes are almost certainly happening in the rainforests of Central Africa, although empirical studies are few. Modern hunting practices in Central Africa are already modifying rainforest ecosystem function, and in synergy with changes in climate and land use may become increasingly influential in the future.

Within this special issue, the future of African rainforests is discussed in relation to likely climate change and land-use scenarios. In this paper, we

— collate and synthesize existing data on direct hunting impacts in the Central African region;
— review the factors driving human hunting in Central African forests and the empirical evidence for indirect ecological impacts of hunting, and discuss how the African rainforests of the twenty-first century are being shaped by current hunting activity; and
— consider how the future scenarios for land-use change (LUC) and climate change outlined in this special issue are likely to influence, and interact with, the drivers of wildlife hunting in Central Africa, to explore the potential long-term consequences for the region's rainforests.

## 2. The hunting footprint

Central Africa is one of the ‘most remote’ areas of tropical moist forest in the world, based on human settlement density, infrastructure and road location [21], yet empirical data from village hunting studies and ecological surveys in the region show that much of this remote forest is already accessed by hunters (table 1).

**Table 1.** Village and commercial hunting distances.

(*a*) evidence of village hunting distances, from village hunting surveys			
source	site	method	maximum distance of traps and hunting camps from village
[22]	Ekom, Cameroon	GPS follow	hunting camps: 18 km
[23]	Oleme and Diba, ROC	GPS follow	traplines: 8.0 (Oleme) and 3.9 km (Diba)
[24]	Ituri, DRC	estimate	most hunting occurs within approximately 15 km of settlements
[25]	Mossapouna, CAR	GPS follow	day hunting: 10 km from village; hunting camps: 21 km from village
[26]	Sendje, Equatorial Guinea	GPS follow	hunting camps: 30 km, hunters trap a max of 3.2 km from the village or a hunting camp (1 day trip)
[27]	Zoulabot Ancien, Cameroon	GPS follow	hunting camps: 21.5 km from village, snares set in a radius of approximately 3 km from camp; every 2 years hunters stay in a hunting camp less than 50 km from the village, for 2 months or more
[28]	Mekas, Cameroon	estimate	village hunting: approximately 5–10 km; hunting camps: approximately 40 km
[29]	Nsiete, Gabon	hunter interviews	more than 10 km in the dry season. Less than 2 km in the wet season
[30]	Midyobo Anvom, Equatorial Guinea	GPS follow	hunting camps: 13.2 km, hunters travelled 2–3 km from hunting camps to place traps
[31]^a^	Dibouka and Kouagna, Gabon	GPS follow	village traplines: 6.5 km; hunting camps 12.7 km
(*b*) evidence of hunting presence in protected areas, from ecological transect surveys			
source	site		furthest record of hunting sign, from nearest road or river (foot transect surveys; in kilometres)
[32]	Nouabale Ndoki, protected landscape ROC		<20
[32]	Ntokou Pikounda forest, ROC		<25
[33]	Mbam Djerem National Park, Cameroon		<27
[34]	Odzala-Koukoua National Park, ROC		<30
[35]	Sankuru landscape, DRC		<40
[36]	Lopé National Park, Gabon		<22
[37]	Mont de Cristal, Gabon		<10
[38]	Lac Tele National Park, ROC		<25
[39]	Waka National Park, Gabon		<10

^a^Maximum trapping distances calculated for this paper; analysis of trapping distances and methods can be found in Coad *et al.* [31].

Humans, as central place foragers, gather food resources in a halo around the village [40,41], and village hunters in Central Africa will usually travel less than 10 km from the village during a day trip (table 1). However, most studies also document village hunters' use of hunting camps. These satellite ‘central places’, situated up to 50 km from the village, are used to catch larger bodied species favoured by the commercial trade [41,42], which are often highly depleted closer to the village where hunting pressure is more intense [31,43]. There have been no direct studies of forest use and offtake by purely commercial hunters in Central Africa: salaried hunters tend not to be based at a fixed point, and their activity is predominantly illegal and therefore concealed. However, ecological transect surveys often record hunting signs such as snares, gun cartridges and hunting paths, which suggests that hunters are penetrating up to 40 km into the forest from the nearest access point such as roads and rivers (table 1).

Even in 1998, more than 40% of Central African forest was within 10 km of a road, and more than 90% was within 50 km of a road [44]. Recent improvements in the road networks of many countries, expansion of logging and other industries and availability of mechanized transport in the region have increased the number of access points for hunters, who are likely to now be accessing the majority of the ‘most remote’ lands throughout the Congo Basin [44,45] (see the electronic supplementary material, figure S1).

## 3. Biological extent and impact of hunting

The biological impacts of hunting comprise both the direct impact on prey species (removal of individuals) and the cascade effects of changing ecological function across the trophic web, as species declining under extreme hunting pressure change their ecological interactions with others [10].

### (a) Prey species

An estimated 178 species are currently hunted and used in the wild meat industry in Central Africa [46]. The survival of over half (97) of these species is deemed threatened by this hunting [47]. Site-specific species lists compiled throughout the region typically show that the majority of local species are used in wild meat consumption and trade, with mammals dominating the harvest (see the electronic supplementary material, table S1). On average, over 60% of village hunting offtake comprises small ungulate and rodent species (see the electronic supplementary material, table S1), which are often caught using wire cable or tough plastic snares. However, escalating gun hunting facilitates the hunting of larger bodied animals, such as elephant or buffalo and primates, which can become the most common taxonomic group at market and village level in regions opened to gun hunting [18,48]. Ivory poaching is currently having exceptionally high impacts in Central Africa [49–51]. Forest elephants across the region declined by 62% between 2002 and 2011 and there is no sign of a fall in the rate of poaching [17]. Almost 90% of elephant carcasses found by guard patrols and on surveys within Central African protected areas in 2011 had been illegally killed [52,53]. Ape populations are also dropping very rapidly in the region, declining by 50% between 1984 and 2000 [54], targeted by hunters for commercial opportunity rather than for family meat [55].

### (b) Changes in wildlife assemblages

Wildlife species are not equally affected by hunting, although some general ecological rules are clear: large, low-density, slow-reproducing and specialist species, such as elephants, will be more vulnerable to increases in predation pressure than smaller, fast-reproducing and high-density generalist species, such as rodents [56,57].

As forest elephants can represent between 33 and 89% of the animal biomass of intact Central African forests, and diurnal primates up to 30% [58,59], the dramatic declines recorded for these species will radically alter functional relationships in which they play a key role [19,60,61]. However, the detrimental and cascading effects of losing large fauna from an ecosystem are not always visible in tropical forests where forest cover and tree density are often used as proxy indicators of ecosystem health [20,62].

**Figure 1.**
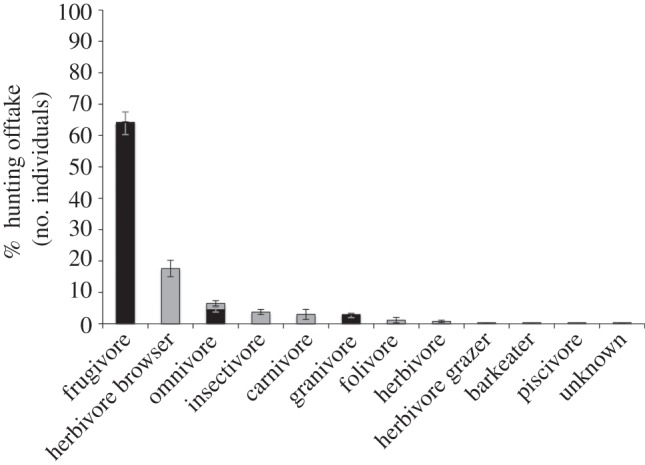
The impact of village hunting on trophic guilds of the Central African forests. Classification of species to these guilds can be found in the electronic supplementary material, table S1. Grey bars represent non-seed disperser, and black bars represent seed disperser.

Although loss of the ‘large forest architects’ may cause the most obvious ecosystem changes, other shifts in species composition will also have important impacts on forest structure and function. Small species released from predation pressure and competition as their natural predators and competitors are hunted to low densities, yet themselves unattractive to human hunters, can find conditions of high hunting pressure favourable and densities may even locally increase, with knock-on consequences for the area's ecology [19,40]. In Central Africa, frugivorous medium-bodied duikers and primates make up a high proportion of hunting offtakes (see figure 1 and electronic supplementary material, table S1), and reductions in their abundance can be dramatic: Lahm [63] recorded local extirpation of yellow-backed duiker around a village of northeastern Gabon less than a year from the onset of the unregulated hunting now typical of the entire region. The role of these species in the middle level of the trophic web, as browsers, seed dispersers and producers of food for the higher trophic levels, makes them a key part of the ecosystem: changes in the abundance of the entire guild are certain to have multiple consequences for the ecosystem [13,19,64]. Leopards, the apex predator in Central African forests, have already been lost from heavily hunted areas due to loss of the species which are their prey base, rather than direct persecution [15].

## 4. Current drivers of hunting

Globally, the need for both protein and income are well recognized as the ultimate drivers of hunting [31,65–67].

Wild meat is a traditional food for almost all ethnic groups in Central Africa, consumed by rich and poor, in urban and rural communities [68,69]. While hunting is predominantly a poor man's activity [70], eating wild meat is not; hunting traditions are a source of pride in urban populations, who pronounce preferences for wild meat and will pay a premium for it, even when their protein requirement can be met with cheaper sources [67,68,71,72]. Vigorous trading of wild meat to satisfy urban demand is widespread in all major Central African cities [71,73–78], and the purchase of wild meat is common in even relatively small towns [41,68,79,80]. The certainty of demand, ease of entering the market and low risk of penalties have encouraged villagers in subsistence economies across the region to use local wildlife as a cash crop [66,81–83].

Commercial hunting has increased markedly in the last 50 years [18,63,81]. Recent studies at village subsistence level have found that more than 90% of resident village hunters sell at least part of their catch in all countries of the region [45,67,81,82,84,85] and that demand along the commodity chain is biased towards larger and rarer species [83,84,86–88]. In addition to village hunters, who are in part hunting to feed themselves, the purely commercial hunters, who often hunt species to order, are accessing more remote land in production forests or protected areas where village land rights do not apply and resources are *de facto* open access [62,69]. This type of hunting is particularly deleterious to the largest species, such as elephants or gorillas, which are typically less highly impacted by village subsistence activities [17,55].

### (a) Sustainability of hunting offtakes

Considerable effort has been made in the last 20 years to develop indices and methods for evaluating hunting sustainability [89–93]. However, contemporary impacts depend enormously on the history of the local area and the dynamic interactions between hunters and the local wildlife community. Impacts of a given hunting pressure can only be predicted in the light of the area's recent past, which has already shaped the communities of both hunters and their available prey [31,94]. None of the sustainability indicators currently in use have been found to perform well [95], and empirical data on wildlife population trends remain the only valid measure of hunting impacts at the species level. To date, using either proxies or direct measures of population trends, only smaller species, such as blue duiker or large rodents, have shown any indication of being resilient to modern Central African hunting regimes sustained over several years [19,29,31,90,96]. If current hunting offtakes are unsustainable, both direct impacts and cascading ecological impacts will intensify over coming years unless hunting practices change.

## 5. Ecosystem function

Ecological systems are shaped by ‘top-down’ forces, such as predation, and ‘bottom-up’ forces, such as climate or land use [10]. The top-down force of overhunting seems already to be causing tropic cascade changes in Central Africa. A suite of empirical studies give evidence for relational changes between species and disruption of ecological function in hunted tropical forests. Although much of the evidence available to date is from outside Central Africa, recent studies in the region confirm similar responses to overhunting to those documented in other tropical forests. Functional changes recorded relate to trophic roles, structural changes in forests, changes in species diversity and richness, seed dispersal, pollination and soil nutrient cycling. Table 2 collates existing empirical evidence for ecological change as a result of hunting, in all tropical forests and African rainforests. Central African forests still harbour large populations of extremely large mammalian ‘forest architects’ or ‘ecological engineers’, such as elephant, hippo, buffalo and gorilla. They are likely to experience significant changes in forest structure after their loss [117]. Importantly, significant implications of mesofaunal loss are also emerging from recent empirical studies [15,19,115].

**Table 2.** Empirical evidence of the impacts of hunting on ecological function in tropical forest ecosystems.

	disruption to ecological functioning as a result of hunting	examples from literature	empirical studies from African rainforests	empirical studies from other tropical forests
changes in wildlife assemblages	interspecific competition (between predator and prey)	apex and mesopredators inhabiting heavily hunted areas are forced to prey on smaller species through direct competition with hunters for larger bodied species; this reduces predator population sizes	[15]	[8,97]
	interspecific competition (between prey species)	heavy hunting pressure and selective hunting can change the competitive balance between sympatric species; many species have separate niches due to competition rather than habitat constraints	[96,98]	[40]
	ecological and predator release	as large herbivores and top predators are extirpated, an initial increase in secondary game species is witnessed; populations of small, non-target species increase but overall animal biomass decreases	[19,79]	[40]
changes in relative seed dispersal success	seed dispersal failure due to seed predators both increasing and switching to more abundant seeds	in heavily hunted areas, rodents and other small seed predators switch feeding behaviour to animal-dispersed species whose undispersed seeds are now found in clusters around the parent plant preference by small-bodied mammal species for small seeds; low predation on large seeds in heavily hunted area	[19]	[99,100]
	reduced seed dispersal, sapling recruitment and plant regeneration	in hunted areas where large, long distance dispersers are absent, rates of dispersal and regeneration of animal-dispersed trees are much lower than where these species are present	[101–103]	[104–107]
	increased kin competition between seedlings	seedlings, which remain under parent plant due to reduced dispersal in hunted areas, experience increased competition		[108]
	changes in relative pre-dispersion predation pressure for seeds	pre-dispersal seed predation by larger mammals is higher in protected areas than in hunted areas		[109,110]
changes in vegetation structure and composition	reduced plant species richness and diversity	tree species richness is lower in regenerating cohorts in hunted areas than in protected sites	[19]	[104,111–113]
	changes in tree spatial structure	increased clustering and densities of saplings of animal-dispersed plants due to loss of large herbivores	[102]	[104]
	increased proportion of lianas (and other wind-dispersed species) due to decrease in animal seed dispersers	wind-dispersed climbing species, which are overwhelmingly woody lianas, are found in higher proportions in the seedling bank at heavily hunted sites than at protected sites	[19]	[106]
	changes in vegetation composition	diversity of plants with large- and medium-sized seeds was significantly lower while diversity of plants with small-sized seeds increased significantly with hunting pressure	[114]	[100]
soil quality	altered nutrient cycling and parasite suppression, disturbed soil fertilization and aeration	reduced abundance of Scarabaeinae beetles as a result of reduced availability of mammal dung alters ecosystem functioning		[113,115]
carbon balance	reduced carbon storage	reduced woody plant recruitment due to decreased large seed species will threaten carbon-storage capacity		[105,116]

Central African hunting systems are biased towards heavy offtakes of seed-dispersing frugivorous mammals; over 70% of animals in an average village hunting offtake have a seed dispersal role (see figure 1 and electronic supplementary material, table S1). Animals disperse the seeds of the majority of tree species in tropical forests [118] and seed dispersal by larger animal guilds particularly affected by hunting plays a key role in both the spatial pattern of tree recruitment and survival [19,61,102,107], and the relationship between tree and liana establishment [104,119,120]. In the absence of specialized dispersing fauna, recruitment and survival of seedlings is reduced for tree species that depend on them [19,121]. Animal-dispersed tropical trees are biased towards species with slower growth, longer life and higher wood density than abiotically dispersed species [105]. A positive (if weak) correlation between seed size and wood density also exists [122]. Animal-dispersed tree species, particularly those with large seeds dispersed by large mammals, therefore contribute a high proportion of the overall carbon-storage capacity of tropical forests. Carbon storage may therefore erode over time if tree regeneration is hampered by changes in faunal guilds, including extinction of large specialized disperser species [61,104,123,124] or increases in seed-predating species enjoying ecological release from their predators [99,125]. Recent data already show significant changes in the seedling layer of hunted West African forests [19,107] with regeneration in defaunated forests favouring faster growing, lower density plants and resulting in overall loss of diversity. This effect has also been recorded in Asian forests [104]. Across the tropics, forests suffering high levels of hunting over the past three decades have also recorded increases in the establishment of fast-growing pioneer species and lianas, and the loss of large hardwood trees [20].

## 6. Impacts of land-use change and climate change on Central African forest structure and function

Central African rainforests will be influenced by both climate change and LUC as well as by hunting over the coming decades. We summarize the climate change and LUC scenarios outlined in this special issue and the recent literature and go on to discuss their potential interaction with the ecological changes caused by hunting, already in motion.

### (a) Land-use change: reduced habitat for forest species, more forests accessed

Central African economies rely on extractive industries, allocating a large part of their territories to formal sector oil, mining, agriculture and extensive timber use [126]. For example, in Gabon, 59% of land is currently allocated to oil and mining production, industrial agriculture and commercial logging, with logging comprising 54% [127]. In 2007, over 30% of all Central African forests were allocated to logging [45]. Despite this allocation, current levels of deforestation in Central Africa are low (0.08% per year from 2000 to 2010, [128]) and driven mainly by increases in population density and subsequent land conversion for small-scale agriculture [128].

Although land conversion and commercial logging are yet to result in large loss of forest canopy cover, the secondary impacts of extractive industries on forests have been substantial. In 2007, logging roads accounted for 38% of the road network in Central Africa, ranging from 13% in DRC to over 60% in Gabon and the Republic of Congo [45], significantly influencing forest disturbance and unregulated human access. Logging infrastructure and industrial roads usher in a domino effect of factors known to intensify hunting pressure, such as population growth from an immigrant workforce [129], increased income and demand for wild meat [130], increased forest access [131–133] and increased extraction to international markets for specialist products like ivory [6,17]. Although logging itself can affect animal densities by modifying habitat at landscape and local scales [134], evidence across the region indicates that secondary impacts of logging activity are currently of far greater ecological importance [135].

Future LUC in Central Africa is difficult to predict beyond the medium term in a region of political and economic instability; however, under current socio-economic conditions, land use may change rapidly, even in the short term [126]. The recent road network expansion in Central Africa, driven by global demand for raw natural resources, is expected to continue to increase access to the remaining remote tracts of forest [45,128], reducing the contiguous forest to smaller blocks both at local scales [136,137] and across the Congo Basin [138]. International demand for agro-industrial products such as oil palm, rubber, sugar cane, coffee and cocoa, is already responsible for forest conversion in the region [128] with governments actively soliciting agro-industrial investment. For example, the government of Gabon recently created a 40/60 joint venture with a large multinational to develop agroindustry as well as other products and services like industrial parks, which ultimately support extractive activities and redistribute worker populations to forest areas [127]. Under scenarios of increasing agro-industrialization and forest access, coupled with intrinsic population growth, unless regulated, hunting offtakes in Central African forests are expected to increase in the short term, and the hunting footprint will be likely to spread into the last remnants of remote forest.

### (b) Climate change impacts: resilient forests?

Climate models for Central Africa, presented in this special issue, suggest that direct impacts of climate change on the region's forest cover may be lower than previously thought by the Intergovernmental Panel on Climate Change. These models predict an increase in temperature, and increased dry signals for Central Africa [139], yet importantly also suggest that extreme precipitation or drought events are unlikely to increase [140,141]. Central African rainforests are also suggested to be more resilient to water deficits compared with moist tropical forests elsewhere [142].

Although extreme change linked to temperature and drying, such as forest die back, seems unlikely for the Central African region [142–144] and devastating forest fires are not predicted in the near future by any of the climate models for Central Africa, even the slight drying associated with higher temperatures, rainfall changes and increased human incursion predicted for this region [128,139,140] may make forests more vulnerable to fire in the future [145,146]. Forest resilience has been used to mean the resistance of the vegetation to change [144]; however, ecological function in even a seemingly ‘resilient’ forest may be significantly affected by the relatively small increases in temperature predicted. Tutin & Fernandez [147] record a guild of trees in Gabon relying on temperature triggers for flowering (and thus fruiting) events. In Uganda, increases in annual temperatures over 30 years have been correlated with a decline in the fruiting and flowering of some tropical tree species, while increasing the fecundity of others [148]. How Central African ligneous species will respond to rapidly changing temperature is poorly understood, but significant impacts on forest species composition or tree productivity could potentially change food availability for animals, affecting animal ranging patterns and densities across the region [149] and initiating trophic cascades as prey distributions, seed dispersal functions and nutrient cycling are in turn changed. These changes would be likely to be additive to any trophic change initiated as a direct result of hunting.

## 7. Discussion

In this paper, we set out to assemble the current empirical knowledge on hunting in Central Africa, its extent, drivers and direct and indirect ecological consequences, and to consider how interactions between hunting and current scenarios for LUC and climate change, outlined in this special issue, might influence the future of the African rainforests.

The data reviewed show that village hunting has been studied in detail at several sites in the six main forested countries of the African rainforest region over the past two decades (table 1). Drivers of hunting are very similar across all countries [65] and direct impacts on species are broadly similar across the Central African forests (see the electronic supplementary material, table S1). Twenty years ago, Barnes *et al.* [150] first documented the deleterious effect of human access on elephant densities in Gabon. Ten years later, Wilkie *et al.* [151] postulated that hunting, rather than habitat loss, would be the greatest driver of wildlife declines across Central African forests, due to increasing road access, and Barnes [152] further predicted that the insidious effects of hunting on wildlife populations would not be realized until species were close to collapse. Barnes' and Wilkie's hypotheses have been upheld for large species [15,17,20,50,51,133]. However, the majority of studies on hunting have focused on hunters, using only proxies for impacts on prey species [88,153]. Data for wildlife population responses to hunting are only available for large species for which robust census methods are available [154,155]. Data on the true status of smaller species, which form the bulk of the wildmeat harvest (see the electronic supplementary material, table S1), are almost entirely absent. Thus, although we have now registered the catastrophic decline of megafauna in the Central African rainforests, the immense consequences of the resulting trophic cascades, on mesofauna densities, forest structure and overall ecosystem function, are only now becoming understood.

As the extent and drivers of the ecological changes underway across the world's ecosystems in the twenty-first century become apparent [9,20,121], research paradigms must widen to become more multidisciplinary. Research on individual impacts of climate change, LUC or hunting on Central African forests risks missing the interactions between these factors. Considering one without the others could lead to widely different conclusions on the future health of Central African rainforests, and conservation, management and research priorities. For example, if viewed solely through the climate change lens, the scenarios outlined in this issue predict minimal change for African rainforests and reasonable resilience to ongoing change [156]. However, coupled with more certain likelihood of LUC, the picture becomes increasingly dynamic and threatening to intact forests and wildlife. LUC predictions, though limited to the medium term, broadly indicate loss of contiguous forest blocks [138], increased accessibility of remote areas, overall forest degradation and increased local populations [128] and permanent agricultural lands [126,128]. The drivers of hunting summarized in this paper are likely to intensify under these LUC scenarios, particularly in regard to the large-bodied, commercially valuable species, which are also more vulnerable to forest cover fragmentation and loss [62,157]. Without greatly improved regulation, intensified drivers will result in intensified hunting offtakes and an increase in the rate of ecological change that these offtakes are effecting. Central African forests now look highly threatened.

Assuming current levels of hunting are maintained, evidence from the studies we reviewed might indicate one possible future scenario for African rainforests: Overhunting severely reduces large ‘forest architects’ and mesofauna, releasing small-bodied seed predators. This promotes fast-growing abiotically dispersed tree species over large-seeded, animal-dispersed, slow-growing, shade-bearing species, reducing carbon storage, fruit availability and associated biodiversity. In turn, the forests' ability to support large frugivorous, seed-dispersing mammals declines, spurring a negative feedback loop whereby both large mammals and large-seeded, long-lived, hard-wooded trees further decline, further reducing carbon storage and thus global resilience to climate change. Although this scenario represents dramatic, permanent change for African rainforests, it may be one plausible, logical outcome that puts the risks of not regulating hunting into perspective.

As Central Africa moves into the twenty-first century, the challenges that LUC and climate change bring are sure to be complex. However, they will be unlikely to reduce the demand for hunting in the region, nor to mitigate any of its ecological impacts. As outlined above, anticipated LUC in the region will probably exacerbate the impacts of hunting and the ecological changes resulting from hunting will probably in turn exacerbate the effects of climate change. LUC and hunting effects are likely to combine to reduce overall carbon-storage potential and biodiversity, diminish overall forest area and, through the structural and compositional changes initiated by hunting, lessen the resilience of remaining forests to drought, and possibly fire, over the long term.

Good hunting management practices and planning are clearly vital to maintaining ecological function in the African tropical forests and must be incorporated into research priorities and overall land-use and climate change strategies [158], as well as impact assessments and private sector management practices on the ground. Conservation practitioners in Central Africa have shown that multiple-use landscapes under efficient management can maintain the large wide-ranging species critical for large tree seed dispersal [159], sustain game populations for hunting needs [160] and support threatened species [161], if a few design rules are applied: for example, including sufficient protected areas and maintaining large timber concessions with a mix of logging histories, including unlogged patches, and ensuring sufficient resources are allocated to controlling hunting and enforcing management plans [160,162]. Similar kinds of landscape-level work are also needed to rethink and reinvent how agriculture might be designed and implemented in Central Africa: for example, to intensify local food production without converting new lands, siting large-scale concessions in already degraded areas, and designing and managing them to mitigate landscape-scale impacts.

The studies on trophic cascades reviewed here (table 2) already indicate that limiting the loss of megafauna and apex predators should become a first priority for conservation strategies that seek to sustain intact ecosystem function in tropical forests [9,121]. However, global demand for ivory, human–wildlife conflicts with megafauna and overall forest loss, coupled with wildlife population declines already suffered, may make retaining viable populations of these species impossible outside large protected areas [51]. Research to determine the responses of mesopredators and ecological niche adjustments, in both wildlife assemblages impacted by overhunting and the plant communities they live in, will be critical in determining the true resilience of the ecosystem and thus in designing hunting management plans and multiple land-use scenarios that could mitigate spiralling and irreversible ecological change.

If future Central African human and wildlife communities are to rely on the range of ecosystem services currently provided by their rainforests—and if the value of these global goods is to be maintained—immediate management of hunting and integration of good hunting practices into large-scale land-use planning must be considered an urgent priority for rainforest preservation and thus an integral and important part of planning for climate change management and mitigation.
